# Automation of 3D liver spheroid generation and acetaminophen dose–response on the MO:BOT enhances assay robustness and precision

**DOI:** 10.1038/s41598-026-58939-4

**Published:** 2026-07-21

**Authors:** Dana Hellmold, Daniel S. Ziemianowicz, Frowin Ellermann, Philipp Depperschmidt, Max Appold, Ahmed S. Omar, Jonathan Kurz, Frank Krieg-Schneider, Thorben Pascal Hoppe, David Hackenberger, Lukas Gaats, Julia Vallverdú

**Affiliations:** mo:re GmbH, Hamburg, Germany

**Keywords:** Modular benchtop 3D cell culture robot, Integrated imaging and liquid handling, Automated liver spheroid generation, Standardized APAP-dose response testing, Reduced spheroid variability, Increased assay robustness, Biological techniques, Biotechnology, Drug discovery, Medical research

## Abstract

Human 3D in vitro models are increasingly adopted in preclinical drug discovery, as they better recapitulate tissue-like architecture, cell-cell interactions, and organ-specific functions compared with conventional 2D cultures. In parallel, initiatives such as the FDA Modernization Act 2.0 and the proposed 3.0 encourage the use of human-relevant in vitro systems as New Approach Methodologies (NAMs) alongside animal models. However, most 3D cell-based workflows rely on manual protocols that are difficult to standardize, producing variable spheroid morphology and fragmented culture steps that impair assay robustness and cross-study comparability. To address these challenges, we present the MO:BOT, a modular benchtop platform designed to standardize and automate complex 3D cell-based workflows. The MO:BOT integrates critical steps of 3D cell culture, including accurate cell seeding, precise medium exchange, on-deck image-based quality control, scalable compound dosing, and downstream assays. Here, we describe a proof-of-concept automated protocol to generate HepG2 liver spheroids and perform acetaminophen (APAP) drug-response testing. Compared with a manual workflow, the MO:BOT not only reduces hands-on time but also minimizes well-to-well variability in liver spheroid size and improves overall spheroid viability. We demonstrate that standardized, well-tuned pipetting routines can maintain the integrity of delicate 3D structures. An MO:BOT-automated APAP dose-exposure experiment with seven concentrations yields a clear sigmoidal cell-toxicity profile with matching changes in viability, LDH release, and ALT activity, demonstrating that improved spheroid uniformity enhances the sensitivity and robustness of downstream assays. The MO:BOT advances the standardization, scalability, and reproducibility of 3D in vitro models, supporting their broader adoption in preclinical research.

**Supplementary Information:**

The online version contains supplementary material available at 10.1038/s41598-026-58939-4.

## Introduction

Human cell-based models have transformed preclinical studies by more closely recapitulating human physiology than traditional animal models^1^. Recent regulatory developments, including the FDA Modernization Act 2.0 and proposed 3.0, explicitly encourage the use of human-relevant in vitro methods as New Approach Methodologies (NAMs)^2–5^ and as alternatives to animal testing. Strategic roadmaps from the FDA and NIH further emphasize the need for qualified, human-relevant models that can deliver reproducible, decision-enabling data in preclinical safety and efficacy testing^4^. As pharmaceutical development increasingly adopts NAMs for early-stage screening and lead optimization, meeting this demand requires platforms that generate reproducible 3D cultures at the level of standardization that regulatory frameworks require.

Among NAMs, three-dimensional (3D) human in vitro models such as organoids and spheroids have emerged as particularly promising alternatives to conventional two-dimensional (2D) monolayers. Unlike monolayer cultures, 3D models recapitulate tissue-like architecture with cell-cell contacts, nutrient gradients, and extracellular matrix interactions^6^. This structural complexity enables improved maintenance of tissue-specific functions and more accurate prediction of human drug responses compared with conventional monolayers^7–9^. Across multiple organ systems, including liver, intestine, kidney, brain, and lung, 3D models have demonstrated the capacity to recapitulate key aspects of human organ physiology and disease states^9^. This physiological relevance positions such in vitro systems as a cornerstone technology for next-generation drug development.

However, widespread adoption of 3D human models faces several challenges that limit their routine implementation. Compared to conventional monolayer cultures, spheroid and organoid systems exhibit greater variability in size and morphology, complicating downstream analysis and reproducibility^10^. In addition, their culture requires more complex and less standardized protocols, as different cell sources, including cell lines, primary cells, and iPSC-derived cells, demand distinct culture conditions and quality control strategies. This complexity is further increased by the variety of culture formats, such as suspension systems, extracellular matrix embedding, or specialized plate-based approaches^7–10^. These workflows are labor-intensive and rely on frequent manual interventions including medium exchange, spheroid monitoring, and quality control. As a result, variability accumulates across handling steps and between laboratories, limiting reproducibility and comparability. Together, the value of 3D in vitro models depends on a common key requirement: achieving robust standardization with consistent size, architecture, and function^11^.

Several approaches have been developed to automate 3D cell culture workflows, but they only partially solve these challenges as they are typically designed for specific tasks rather than end-to-end workflows. Liquid handlers increase scalability for downstream analysis but do not address the upstream generation of homogeneous 3D models or accommodate the diverse handling requirements of different culture types^12,13^. 3D bioprinters support the creation of complex, ECM-embedded constructs but typically focus on specific applications and lack flexibility for suspension cultures, different plate formats, or long-term maintenance^14–16^. Bioreactors are focused on scaling up the production of 3D cultures but often generate heterogeneous aggregates with variable size and viability, and lack the precise control needed for compound screening^17,18^. Organ-on-a-chip systems provide physiologically relevant, dynamic culture conditions but are limited by low throughput^19,20^. Microwell and microfluidic platforms enable controlled spheroid formation but remain application-specific and do not integrate the full workflow^21,22^. A dedicated, integrated platform specifically designed to address 3D cell culture standardization is missing. Such a system needs to reduce operational complexity and provide flexibility to accommodate multiple cell sources, model types, culture formats, and downstream applications. It should unify spheroid generation, long-term maintenance, perturbation, and readout within a single device^11^.

To address these needs, we developed the MO:BOT, a benchtop automation platform designed to standardize all operator-dependent steps of complex 3D cell-based workflows. The MO:BOT integrates critical steps of 3D cell culture: (1) spheroid and organoid generation and long-term maintenance, (2) controlled medium exchange, (3) compound dosing, (4) imaging-based quality control, and (5) endpoint assays, within a single enclosed system. The platform combines modular, plug-and-play on-deck components (heater, cooler, tilter, shaker, imaging module, and tip, plate, and tube holders) to support diverse 3D culture workflows and applications, while minimizing plate transfers, and reducing user-dependent variability and manual interventions. The MO:BOT software provides an intuitive, integrated protocol library covering multiple human 3D in vitro models, and enables straightforward implementation of new, customized workflows.

In this work, we demonstrate the MO:BOT’s capability using a liver spheroid generation and toxicity workflow as a proof-of-concept application. As a model system, we employed HepG2 cells cultured in 3D, a well-characterized human hepatocarcinoma cell line that exhibits enhanced functionality and improved physiological relevance compared to monolayer cultures^14,23^. Moreover, HepG2 spheroids are widely used in early-stage screening due to their robustness and compatibility with high-content platforms^24^. We show that the MO:BOT enables standardized and scalable generation of liver spheroids suitable for compound screening. This is achieved by combining the biological advantages of 3D liver models with the process-level benefits of automation. This approach provides a path toward high-quality, robust in vitro assays aligned with modern, human-relevant drug development and with emerging regulatory expectations under initiatives such as the FDA Modernization Act 2.0 and proposed 3.0^2–5^.

## Results

### The MO:BOT enables a 3D-specific automated workflow for liver spheroid generation and toxicity testing

**Fig. 1:**
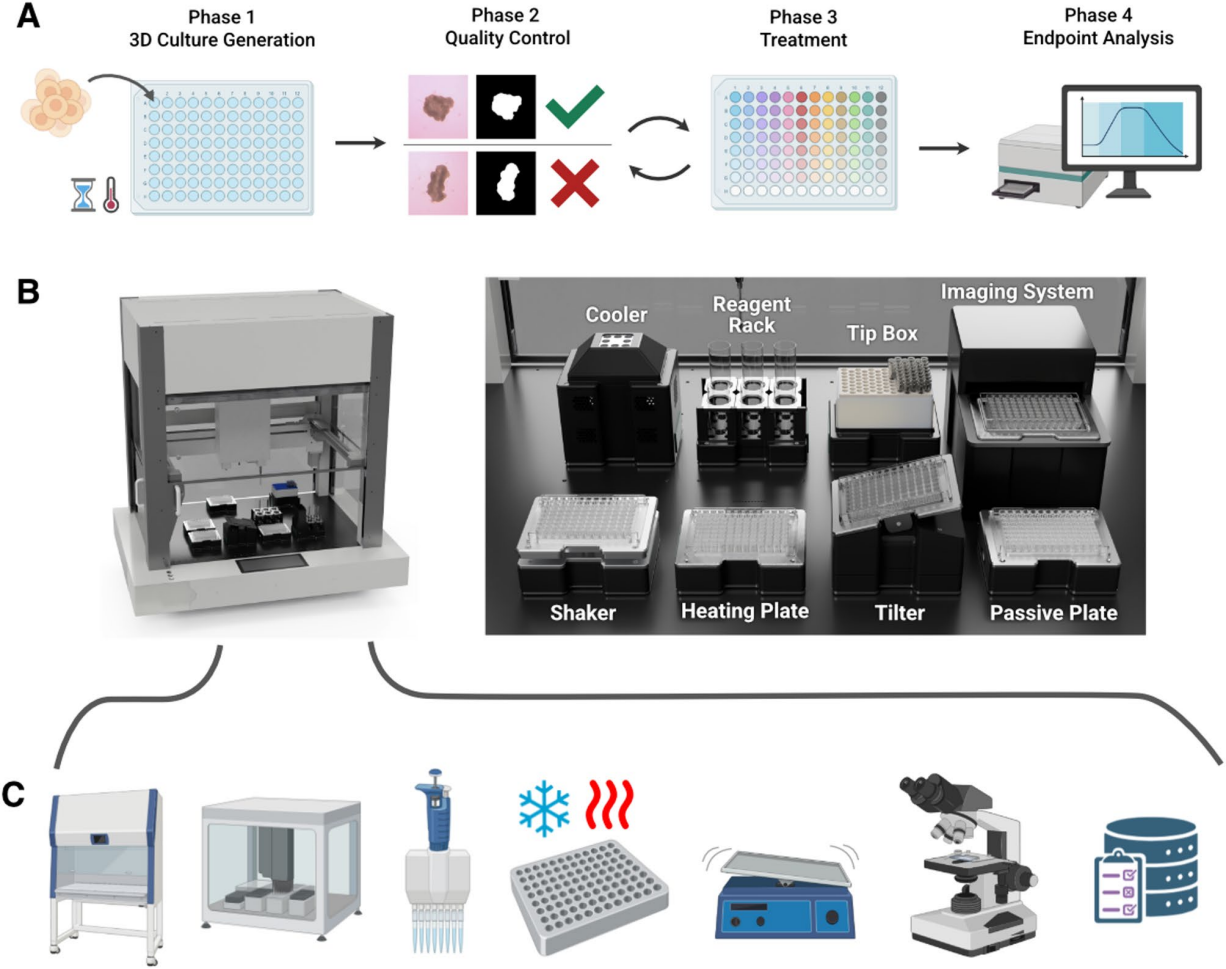
The MO:BOT, an integrated automation platform that consolidates the traditional cell culture workflow. (**A**) A typical 3D cell culture workflow occurs in four phases: 1) cell seeding and maintenance of the culture over days or weeks, wherein  2) quality control is performed throughout to track growth and development, followed by 3) treatment by e.g. stimulation with a small molecule and additional 2) quality control and terminated by 4) an endpoint assay. (**B**) Cell culture workflow integration in the MO:BOT. The MO:BOT consolidates all steps with interconnected on-deck modules. This enables e.g. temperature control during pipetting steps and seamless data collection via brightfield imaging. (**C**) Traditional workflows require transfers between separate instruments (e.g. biosafety cabinet, liquid handler, shaker, plate reader, microscope etc.), introducing sterility risks, time variability at each transfer step, increasing overhead and does not allow for systematic progress tracking and data collection.

The MO:BOT executed all key phases of 3D spheroid culture within a single, integrated platform (Fig. 1A). A typical 3D cell culture experiment comprises four phases1) cell seeding and maintenance of the culture over several days or weeks,^2^) iterative quality control to monitor spheroid growth and morphology,3) treatment with a compound such as acetaminophen (APAP), again followed by intermediate quality control, and finally 4) termination of the culture by performing endpoint assays such as for viability, cytotoxicity, and functionality.

The hardware architecture of the MO:BOT is designed specifically around 3D culture handling rather than generic liquid handling. A configurable set of on‑deck modules, including shaker, heating plate, cooler, tilter, reagent rack, tip box, passive plate positions, and an integrated imaging system, are arranged within a single enclosed workspace to support both spheroid maintenance and treatment steps **(**Fig. 1B, and Supplementary Fig. 1). This plug-and-play modular design enables users to reconfigure MO:BOT modules according to the specific requirements of each workflow. By bringing functions that are typically distributed across multiple instruments, such as manual pipetting, plate handling, shaking, temperature control, imaging, and assay preparation, onto one dedicated 3D culture platform, the MO:BOT automates the essential workflows of a conventional 3D cell culture within a unified device **(**Fig. 1C**).** This 3D‑centric integration is expected to support standardized spheroid handling, reduce operator‑dependent variability in timing and manipulation, and provide a scalable basis for robust automated 3D culture workflows.

### Automated cell seeding with MO:BOT yields uniform liver spheroids

**Fig. 2:**
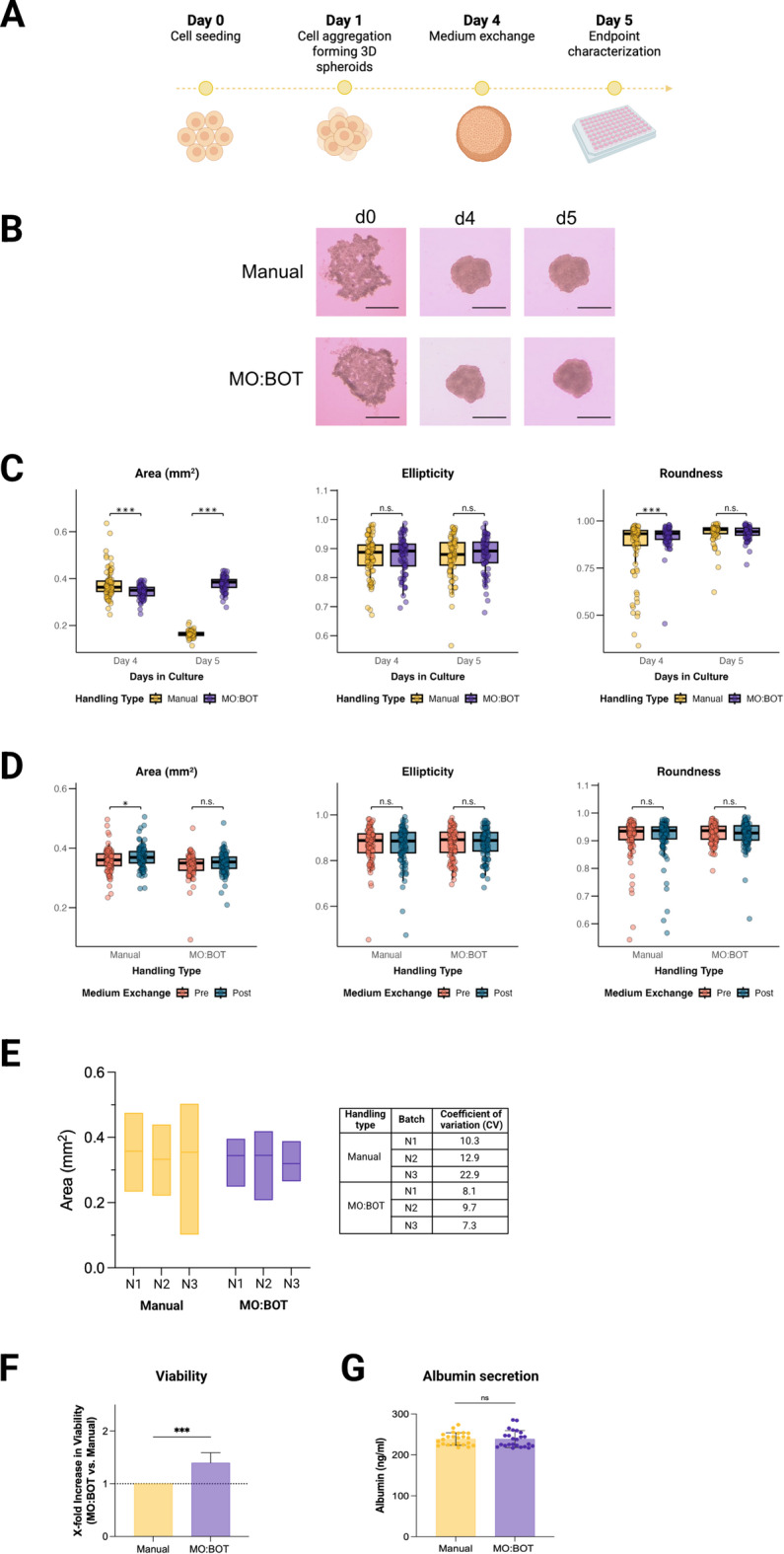
Automated spheroid generation reduces morphological variability and enhances viability without modifying culture protocols. (**A**) Experimental design for HepG2 liver spheroid formation and characterization performed with both handling methods, Manual and MO:BOT. (**B**) Representative sample of brightfield images collected with the MO:CROSCOPE at day 4 and 5 of culture (scale bars: 500 μm). (**C**) Time-course analysis reveals significant differences in area between handling methods, prior to medium exchange, at day 4 and 5, with comparable shape metrics (ellipticity, roundness). *n* = 120 for each day and each handling method. (**D**) Medium exchange on day 4 shows little or no effect on spheroid morphology, regardless of handling type. *n* = 96 for each handling method. (**E**) MO:BOT achieves significantly lower coefficient of variation in spheroid area across independent experimental runs compared to manual pipetting on day 4 (*p* < 0.001). *n* = 96 per batch. (**F**) Liver spheroids on day 5 has a higher viability than ones generated manually). *n* = 36 per handling method. (G) Liver spheroids generated with both handling conditions present an equivalent albumin secretion. *n* = 24 per handling method.

To benchmark automated spheroid generation against the established manual workflow, we implemented an automated protocol in the MO:BOT software that covers cell seeding, spheroid formation, medium exchange, and endpoint characterization, as schematized in Fig. 2A. Brightfield imaging acquired with the integrated MO:CROSCOPE on days 0, 4, and 5 demonstrated robust spheroid formation under both conditions, with compact, well-defined aggregates visible by day 4 and maintained at day 5 (Fig. 2B). No qualitative differences in overall shape or edge definition were observed between manual and MO:BOT spheroids, indicating that the automated seeding and maintenance workflow supports efficient spheroid self-assembly and yields uniformly compact aggregates. We then performed MO:CROSCOPE-based quantitative analysis of morphological parameters (area, ellipticity, and roundness) to assess how automation influences spheroid size and shape over time. From day 4 to day 5, the average spheroid area decreased from 0.37 mm² ± 0.05 to 0.16 mm² ± 0.01 in the manual workflow, whereas spheroid area remained stable in the automated workflow (Fig. 2C), suggesting that automated handling stabilizes spheroid size during liver spheroid maintenance. Ellipticity and roundness values stayed constant across all groups and time points, indicating that neither handling type nor culture duration measurably affected spheroid shape (Fig. 2C).

As medium exchange is a critical and potentially disruptive step in 3D culture, we evaluated whether the automation of this procedure affects spheroid architecture by comparing morphological metrics before and after the full medium exchange step performed on day 4 (Fig. 2D). Quantitative analysis of morphological parameters before and after exchange revealed a decrease in spheroid area for the manual workflow, whereas area remained unchanged for MO:BOT handling, with no significant differences in ellipticity or roundness for either condition. These data indicate that the automated medium exchange routine is sufficiently gentle to preserve size and overall spheroid structure. To directly assess spheroid uniformity, we next compared spheroid area and variability at day 4 between both workflows and experimental runs. While the median spheroid area was comparable between conditions, MO:BOT cultures achieved a markedly lower coefficient of variation (CV) in spheroid area (8.3%) than manual cultures (15.3%) (Fig. 2E), demonstrating that the automated workflow produces highly uniform spheroid populations. Moreover, analysis of coefficient of variation across independent experiments revealed consistently lower CV values for MO:BOT than for manual **(**Fig. 2E**)**, indicating reduced inter-batch variability in spheroid size. Such reduced intra- and inter-experiment variability is expected to benefit downstream toxicity testing, where assay sensitivity and statistical power depend on consistent baseline morphology.

Finally, to confirm that these morphological findings translate into preserved cellular function, we assessed the viability and albumin secretion of HepG2 spheroids at day 5 of culture. Area-normalized viability expressed as a fold change relative to manual handling, was 1.4-fold higher for MO:BOT spheroids (Fig. 2F), indicating that automated handling not only preserves but slightly improves overall spheroid viability. Albumin secretion per spheroid was not significantly different between manual and MO:BOT cultures (Fig. 2G). Collectively, these results demonstrate that automated spheroid generation with the MO:BOT yields liver spheroids with functional characteristics comparable to manual culture, while substantially reducing variability in spheroid morphology and increasing size uniformity and viability, thereby providing a robust foundation for the subsequent toxicity study.

### Automated acetaminophen (APAP) dosing induces concentration-dependent toxicity in liver spheroids

**Fig. 3:**
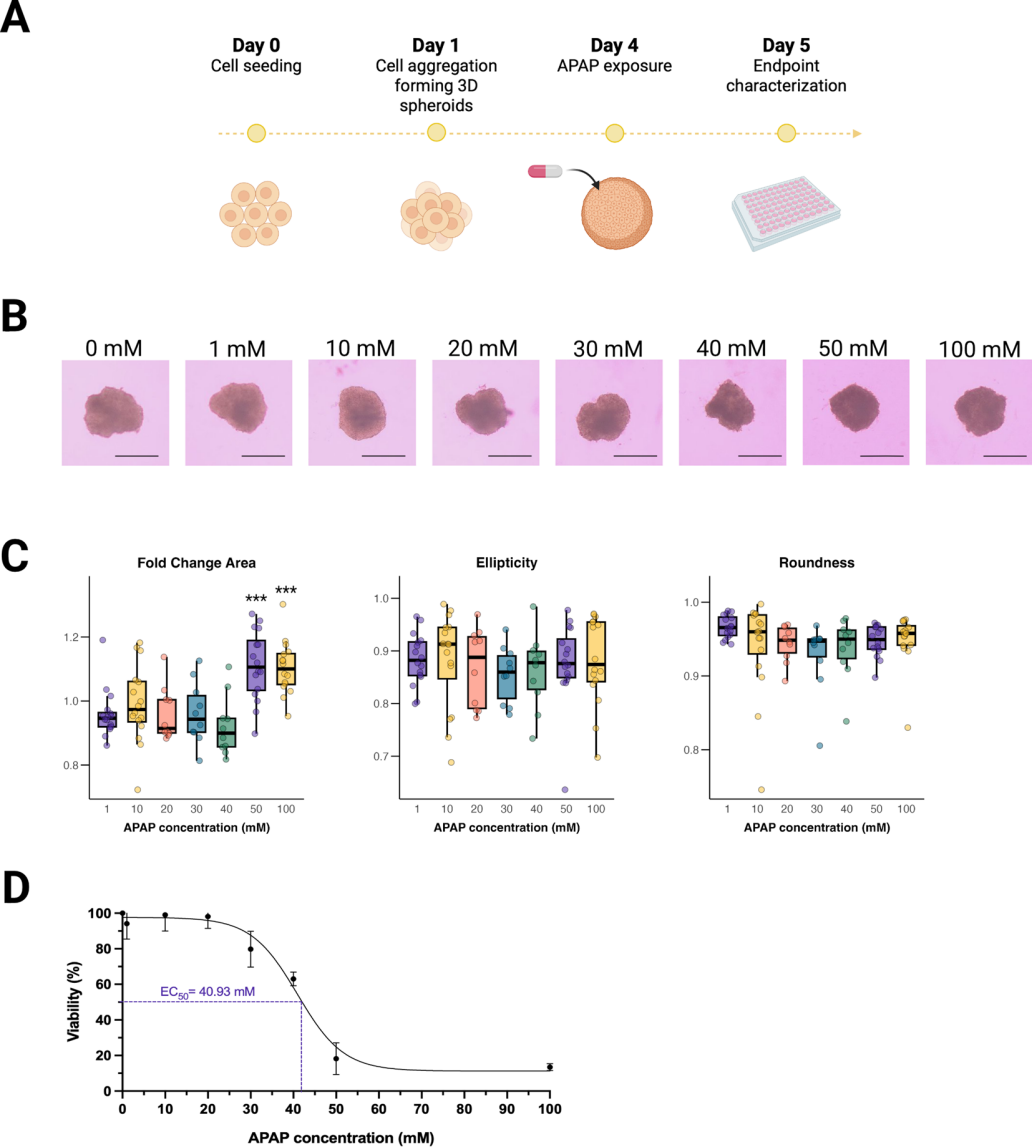
The MO:BOT enables cell toxicity screening. (**A**) Experimental design for dose-response assessment; APAP exposure on day 4 after seeding. (**B**) Representative brightfield images, collected with the MO:CROSCOPE, of spheroids exposed to a range of APAP concentrations show no obvious morphological disruption (scale bars: 500 μm). (**C**) Morphological analysis reveals a dose-dependent increase in spheroid cross-sectional area at 50 mM and 100 mM APAP concentrations, while ellipticity and roundness show no significant differences across the concentration range. *n* = 16 per APAP concentration. (**D**) The viability dose-response curve yields an EC₅₀ of 40.93 mM. *n* = 16 per APAP concentration.

To assess cell toxicity under automated conditions, we integrated an acetaminophen (APAP) exposure routine into the MO:BOT workflow, adding the compound on day 4 and performing endpoint characterization on day 5 (Fig. 3A). Spheroids generated with the MO:BOT were treated with a concentration range of 0 to 100 mM APAP, enabling quantitative assessment of both morphological and functional responses to APAP.

Brightfield imaging at day 5 revealed no gross structural disruption of spheroids across most APAP concentrations, but a concentration-dependent change in optical appearance (Fig. 3B). At concentrations up to 30 mM, spheroids remained compact with well-defined sharp edges in the MO:CROSCOPE 2D projection images, and transparency comparable to untreated controls. From ≥ 40 mM APAP, spheroids appeared progressively less transparent, and at 100 mM the spheroid borders became less well defined (“fuzzy”), indicating subtle structural alterations.

Automated quantitative image analysis of spheroid morphology at day 5 using the integrated MO:CROSCOPE confirmed that APAP exposure only modestly affected area (Fig. 3C). Fold-change analysis of spheroid area relative to untreated controls revealed a significant increase in size at 50 mM and 100 mM APAP compared with all lower concentrations, whereas spheroids treated with ≤ 40 mM remained close to baseline. Ellipticity and roundness did not exhibit any dose-dependent changes across the tested concentration range, indicating that overall spheroid shape was largely preserved despite these high-dose-induced size increases (Supplementary Fig. 2).

In contrast, functional readouts showed a clear concentration-dependent toxicity profile in the spheroid model (Fig. 3D). Fitting the ATP-based viability data with a four-parameter sigmoidal model yielded an EC₅₀ of 40.93 mM APAP, which we used as the target concentration for subsequent experiments. Viability remained close to control levels at low concentrations (≤ 20 mM), started to decline at intermediate doses (around 40–50 mM), and approached near-complete loss of viability at 100 mM APAP. These results show that the MO:BOT-based assay detects a clear, quantitative APAP toxicity profile with a well-defined EC₅₀, while maintaining overall spheroid morphology across the tested dose range.

### Automated APAP toxicity study at EC₅₀ preserves spheroid integrity while capturing toxic effects

**Fig. 4:**
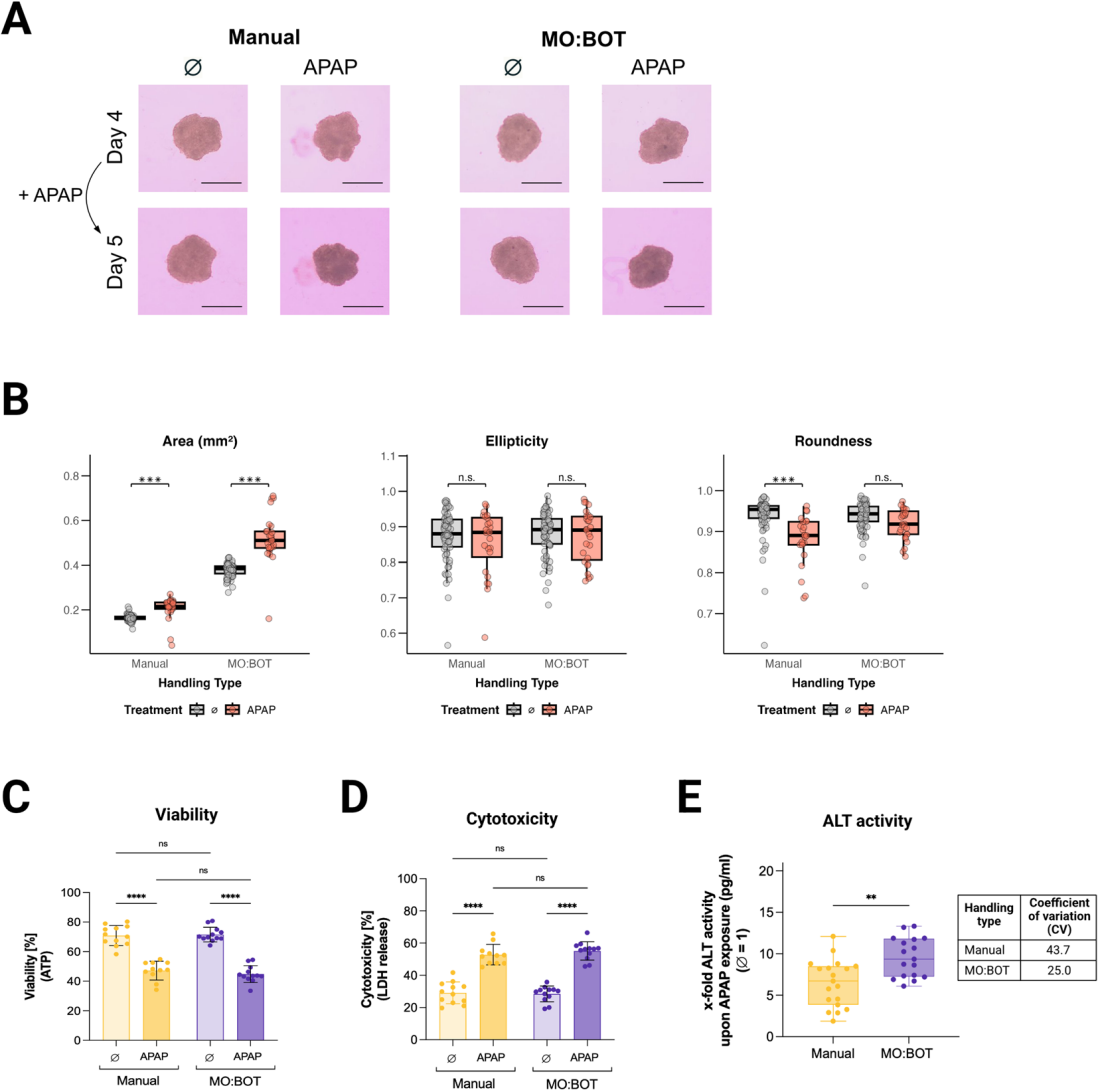
The MO:BOT-automated handling enhances sensitivity and reduces variability in hepatotoxicity detection. (**A**) Representative brightfield images showing spheroid morphology before and 24 h after 40.93 mM APAP treatment with EC_50_ 40.93 mM for both handling methods (scale bars: 500 μm). (**B**) Morphological analysis reveals APAP-induced increase in spheroid area (*p* < 0.001) with preserved shape metrics, independent of handling methods. *n* = 72 per handling method and treatment. (**C**,** D)** Viability decreases and cytotoxicity increases (*p* < 0.0001) independently of handling type. *n* = 12 per handling method and treatment. (**E**) MO:BOT-generated spheroids exhibit significantly greater ALT release upon APAP treatment compared to manually handled spheroids (*p* < 0.01), indicating enhanced sensitivity for detecting hepatocellular injury. *n* = 18 per handling method.

To evaluate cellular responses at the previously established EC₅₀ concentration, we exposed liver spheroids to 40.93 mM APAP on day 4 of culture and, on day 5, compared spheroids generated and treated with manual or MO:BOT handling. Pre-treatment brightfield images showed compact, well-defined spheroids for both workflows and both conditions, with no visible differences between groups. After 24 h APAP exposure, spheroids retained their overall shape but displayed slightly reduced transparency compared with untreated controls in both handling types (Fig. 4A).

APAP treatment increased spheroid area in both workflows, with MO:BOT spheroids remaining larger than manually handled spheroids (Fig. 4B). Ellipticity was unchanged across conditions, indicating that APAP exposure and handling type did not affect overall spheroid elongation. Roundness decreased slightly after APAP exposure in both workflows, a minor effect that did not compromise spheroid integrity or assay suitability.

To examine whether automated handling affects the functional readouts at the EC₅₀ APAP concentration, we compared viability and cytotoxicity between manual and MO:BOT workflows. Viability decreased to 47% in manually handled spheroids and to 45% in MO:BOT spheroids, and cytotoxicity increased to 53% and 55%, respectively. These differences were not statistically significant (Fig. 4C and D). These findings indicate that automated handling preserves the sensitivity of 3D liver spheroids to toxic treatment.

To determine whether cell-injury-associated markers were similarly captured under both handling conditions, we next quantified alanine aminotransferase (ALT) release in response to APAP exposure. ALT activity increased upon APAP treatment in both workflows, consistent with APAP-associated cellular injury in the liver spheroid model **(**Fig. 4E**).** The increase in ALT was significantly more pronounced in MO:BOT spheroids than in manually handled spheroids, and variability was lower in the automated condition (CV 25.0% vs. 43.7% for manual). This combination of higher signal and reduced variability suggests that the more morphologically uniform spheroids produced by the automated workflow may provide a more sensitive and robust ALT readout in liver spheroids.

## Discussion

In this study, we present the MO:BOT, a dedicated automation platform for 3D cell culture workflows, and demonstrate its capacity for standardized liver spheroid generation and toxicity testing using HepG2 spheroids as a proof-of-concept model. Our results show that the MO:BOT reliably automates all key steps of 3D cell culture experiments, comprising cell seeding, medium exchanges, image-based quality controls, compound dosing, and endpoint assays within a single integrated system. Importantly, the platform maintains and improves the biological performance compared to manual handling in terms of spheroid morphology, viability, and functional output.

The MO:BOT’s integrated workflows are designed to address critical prerequisites for the broader adoption of 3D human cell-based models in preclinical research: the standardization, reproducibility, and consistency of these in vitro models. Inconsistent spheroid size and morphology across wells, plates, and experimental batches remain a major barrier to implementing 3D models in drug development, as they introduce variability that complicates data interpretation and limits cross-laboratory comparability^11^. Standardized generation of uniform spheroid populations is therefore essential for both assay performance and qualification of 3D models as NAMs^2–5^. Our data demonstrate that the MO:BOT workflow substantially reduces spheroid size variability compared to manual handling in both inter- and intra-batch experiments. Moreover, automated medium exchanges maintain a stable spheroid area, while manual exchanges cause measurable size reduction. These improvements are directly attributable to the pipetting system, which standardizes seeding volume, dispensing speed, and aspiration depth based on cell source, medium viscosity, spheroid size, and plate format. While existing automated platforms address individual workflow steps such as seeding, imaging, or reagent exposure, they typically require multiple instruments and inter-device transfers that reintroduce variability^12–15,17–22,25^. The MO:BOT integrates the four critical cell culture steps within a single enclosed platform, eliminating these handling discontinuities and the associated variability from repeated plate transfers, environmental exposure, and timing inconsistencies.

Equally important to morphological standardization is the capacity of the automated workflows to preserve the cell phenotype and functionality, ensuring the applicability of these in vitro models for disease modeling studies, toxicity, and drug screening assays^7,8^. In this study, HepG2 spheroids generated and maintained with the MO:BOT demonstrated functional characteristics fully comparable to manually handled spheroids, validating the biological integrity of the automated workflow. Albumin secretion did not differ between the two workflows, consistent with reports that HepG2 spheroids maintain liver-specific protein synthesis capacity across different culture platforms^26,27^. Notably, area-normalized viability was 1.4-fold higher in MO:BOT spheroids compared to manual handling, likely due to the automated pipetting strategy that minimizes shear-stress-induced cell damage during liquid handling steps. Studies have demonstrated that pipetting-induced shear forces can significantly reduce cell viability and trigger stress-activated signaling pathways, with effects becoming evident up to 24 h post-handling^28,29^. These results confirm that automation-driven improvements in morphological standardization translate into preserved and slightly enhanced cellular function, providing a robust foundation for downstream compound testing.

Having established the quality and reproducibility of MO:BOT-generated spheroids, we next addressed a critical bottleneck in the adaptation of 3D models for preclinical applications: the need for scalable screening workflows. For this, we executed a complete, multi-step cell toxicity workflow using acetaminophen (APAP) as a well-characterized benchmark hepatotoxin. This proof-of-concept study assessed whether the MO:BOT can transition from standardized spheroid generation to compound screening applications while maintaining the morphological uniformity and biological integrity established during culture. The ATP-based viability assay revealed a clear sigmoidal dose-response profile with an EC₅₀ of 40.93 mM, in line with reported APAP EC₅₀ values for HepG2 spheroid models under similar culture conditions. This dataset confirmed that the automated workflow preserves the expected toxicological responses^30,31^. Notably, bulk spheroid morphology remained largely preserved across the APAP dose range, while functional and injury readouts changed markedly. This pattern, where functional damage appears before structural breakdown, is well-documented in 3D liver models and highlights the value of integrating multiple complementary endpoints in spheroid-based toxicity testing^31^.

At the established EC₅₀ concentration, viability and cytotoxicity values were equivalent between automated and manually handled spheroids. Critically, the automated workflow demonstrated a clear advantage in the ALT release assay: MO:BOT spheroids exhibited a more pronounced ALT increase upon APAP treatment and substantially lower inter-replicate variability compared to manual handling. As a sensitive marker of hepatocellular injury, ALT release is directly dependent on spheroid integrity and metabolic consistency across the population^16,25,32^. The improved signal magnitude and reduced variability are therefore a direct consequence of the superior morphological uniformity achieved by the automated workflow; more consistent spheroid size and architecture produce a more homogeneous biological response to compound exposure. These findings demonstrate that the MO:BOT not only scales up the 3D culture workflow, but also actively enhances the sensitivity and statistical power of functional injury readouts, a key advantage for hepatotoxicity screening where reliable detection of subtle, early-stage toxicity signals is essential.

It is important to acknowledge that HepG2 cells, while widely used in early-stage screening due to their robustness and ease of culture, exhibit limited metabolic competence compared to primary hepatocytes or iPSC-derived hepatocytes, particularly with respect to CYP450-mediated biotransformation^17,20,24^. Consequently, the APAP concentrations required to elicit a toxic response in HepG2 spheroids are higher than those observed in more physiologically representative models, which is a recognized limitation of this cell line^19,20,24^. HepG2 spheroids were selected deliberately as a well-characterized, reproducible system to validate workflow automation, not to establish a clinically predictive assay. Future applications with more complex 3D liver models, such as primary and iPSC-derived hepatocytes, can be readily implemented on the MO:BOT thanks to its flexible and interchangeable modular design.

The MO:BOT directly bridges the gap between the biological promise of 3D human in vitro models and their practical implementation as NAMs in regulatory-relevant drug development settings. Current regulatory roadmaps from the FDA and NIH explicitly identify reproducibility, standardization, and scalability as key requirements for the qualification of in vitro models as NAMs^2–5^. The MO:BOT directly addresses these requirements by integrating spheroid generation, maintenance, perturbation, imaging, and endpoint analysis within a single, standardized platform. The reduced inter and intra-batch variability demonstrated here is particularly relevant in this context, as it supports the generation of consistent, decision-enabling datasets that can be compared across experiments, laboratories, and platforms. The modular architecture of the MO:BOT is designed to accommodate this expansion, toward primary spheroids, patient- and iPSC-derived organoid models, and multi-organ systems, as the field moves toward formal NAM qualification^2–5^.

## Methods

### MO:BOT, the 3D cell culture automated device

The MO:BOT, a modular benchtop automation system developed by mo:re GmbH, was employed for the automated generation of 3D human liver spheroids and subsequent toxicity studies in high-throughput 96-well plates. This platform integrates multiple interchangeable plug‑and‑play modules, without the need for cables or screws, across ten configurable work fields to support specific cell culture workflows. The system includes the following functional modules: 1) MO:CROSCOPE, a brightfield imaging unit used to collect and analyze morphological parameters of individual 3D cell aggregates, 2) MO:HEAT, a heating module capable of reaching up to 40 °C for applications such as sample incubation, enzymatic reactions, and controlled handling of temperature-sensitive matrices (e.g., ECM polymerization), 3) MO:COOL, a cooling module capable of maintaining temperatures down to − 5 °C to ensure thermal stability for temperature sensitive experiments, such as handling and dispensing of ECM hydrogel for embedded 3D models, as well as short‑term sample storage, 4) MO:TILT, a tilting module enabling gentle mixing to enhance medium exchange and homogeneous distribution of cells and reagents, and 5) MO:SHAKE, a shaker operating up to 1,000 rpm for vigorous mixing and ensuring reaction uniformity. Additionally, the MO:BOT features several passive modules: 1) MULTIRACK, a reagent rack compatible with various tube sizes ranging from 1.5 mL to 50 mL, 2) TIP BOX MODULE, used to register the placement of pipette tips of different volumes such as 250 µL and 1000 µL, and 3) PLATE HOLDER, a module designed to accommodate various plate formats (U-Bottom, V-Bottom, F-Bottom plates) and sizes from 6-well to 384-well plates. The MO:BOT can be equipped with single- and multi‑channel positive‑displacement pipettes capable of handling both viscous and non‑viscous liquids, enabling precise cell seeding, medium exchange, and compound dosing to promote consistent culture conditions. To ensure optimal performance across diverse 3D culture protocols and applications, the pipette system undergoes a comprehensive four parameter calibration process: 1) Volume calibration ensures accurate dispensing across the operational range (dependent on tip size), minimizing volume-dependent errors; 2) Pipetting strategy calibration optimizes aspiration and dispensing routines for both multi-dispensing and single-dispensing modes, accommodating different reagent types and workflow requirements; 3) Plate-to-tip positioning calibration precisely maps the spatial relationship between pipette tips and target wells for each plate format (e.g., 96-well, 48-well, U-bottom, V-bottom, and F-bottom); and 4) Dynamic model protection calibration adjusts aspiration depth and speed based on spheroid size, creating a protective zone above settled aggregates to prevent mechanical disruption during medium exchange while maximizing supernatant removal efficiency. This multi-parameter calibration framework enables gentle, reproducible handling of delicate 3D structures while maintaining high-precision liquid transfer across all workflow steps. In addition, a robotic gripper enables automated plate transfer between modules within the workspace.

For the present study, the integrated single‑channel pipette, MO:CROSCOPE (QM, mo:re GmbH), Plate holder (HM, mo:re GmbH), Tip box module (PM, mo:re GmbH), and Reagent rack (MM, mo:re GmbH GmbH) were utilized. The “Liver spheroids generation and toxicity testing” protocol, available in the MO:BOT software (moreOS), was used for both spheroid formation and subsequent drug exposure. The protocol comprises three routines executed at defined time points: day 0, cell seeding, day 4, medium exchange with or without the acetaminophen (APAP), and day 5, sample collection or endpoint analysis. As part of the day 4 and day 5 routines, brightfield images of each well were acquired and analyzed using the MO:CROSCOPE immediately before and after medium exchange. In addition, the protocol “Liver spheroids APAP dose titration” comprising two routines, cell seeding and medium exchange, was performed to determine the APAP EC_50_.

Upon protocol initiation, the system provides step-by-step guidance for the user to correctly place modules, labware, and reagents across designated work fields within the MO:BOT platform. Following this setup, each module is automatically recognized and activated by the system. Automated pipetting is then performed with high precision and accuracy to ensure uniform cell distribution and consistent reagent handling. Data generated by the MO:CROSCOPE module are used to monitor and analyze spheroid morphology and culture evolution throughout the experimental process. Further details about the MO:BOT system are provided in the Methods sections, “Automated liver spheroid generation”, “Automated acetaminophen (APAP) dose titration”, and “Automated Medium Exchange for EC50 APAP stimulation”, as well as in Supplementary Fig. 1, Supplementary Video and at https://more.science.

### Liver cell culture

HepG2 cells (ATCC, HB-8065) were maintained in Dulbecco’s Modified Eagle Medium (DMEM) (DMEM high glucose, Sigma-Aldrich, D0819-500ML) supplemented with 10% Fetal Bovine Serum (FBS) (Thermo Fisher Scientific, A5670701) and 1% Penicillin–Streptomycin (P/S) (Thermo Fisher Scientific, 15140-148), hereafter referred to as DMEM complete medium. Cells were incubated at 37 °C and passaged at 70% confluency using Trypsin-EDTA Solution (Himeda, TCL033) to maintain exponential growth. Cells were routinely screened for mycoplasma contamination using a PCR-based detection assay (Thermo Fisher Scientific, M7006), and all cell cultures tested negative throughout the study.

### Automated liver spheroid generation

For all experiments, HepG2 cells were used within a defined low passage range (passage 18–25), and the same passage number was applied across biological replicates and assay runs to ensure consistency. Before seeding, cells were harvested, counted using a hemocytometer, and resuspended in complete DMEM medium at the desired concentration. Cell seeding was performed with the MO:BOT, and an additional plate was seeded manually to evaluate the impact of automated handling on spheroid quality and uniformity.

For automated spheroid generation, the MO:BOT protocol “Liver spheroids generation and toxicity testing”, routine 1, was selected from the integrated protocol library. Required consumables were positioned on the corresponding mo:re modules (described in the Methods section “MO:BOT, the 3D cell culture automated device“), including a 96-well U-bottom ULA plate (FaCellitate, F202003), a 15 mL tube containing the prepared HepG2 cell suspension, and a box of 1,000 µL positive-displacement tips (mo:re GmbH, MO: RE 1000 µL piston tips), and placed on the MO:BOT work field. Upon protocol initiation, the MO:BOT’s single-channel pipetting tool automatically picked up a tip, aspirated the defined volume of cell suspension, and dispensed 2,000 cells in 150 µL of medium per well to support uniform spheroid formation (^32^). After seeding under both handling conditions, plates were transferred to a humidified incubator at 37 °C, 5% CO_2_ for 4 days to allow spheroid aggregation.

### MO:CROSCOPE, automated brightfield imaging module

Spheroid morphology was assessed using the MO:CROSCOPE imaging system integrated into the MO:BOT. Cell culture plates were loaded into the MO:CROSCOPE carrier, and brightfield images were acquired under standardized illumination and focus settings using a 5X ZEISS A-Plan objective, IMX477 sensor (Arducam B0279) with an image resolution 2160 × 2160 pixels (center-cropped from full sensor) and pixel size of 0.79 μm/px. The MO:CROSCOPE automatically captured brightfield images of each individual spheroid across a full 96-well plate and analyzed in real-time key morphological parameters, such as size, roundness, and ellipticity. Imaging of a full 96-sample plate occurred within 10 min, limiting the time of the plate outside of the incubator. The MO:CROSCOPE served as a quality control check for spheroid growth and was used before and after medium exchange and drug stimulation to monitor potential morphological changes. Area, roundness, and ellipticity were analyzed for *n* = 120 spheroids per day and handling method in the characterization study, *n* = 16 spheroids per APAP concentration in the dose-response study, and *n* = 72 spheroid per handling method and treatment in the APAP EC_50_ study.

### Viability assay

To assess spheroid quality, cellular ATP content was measured using the CellTiter-Glo^®^ 3D Cell Viability Assay (Promega, G9681). The assay was performed following the manufacturer’s instructions. For each handling condition, 50 µL of culture supernatant containing the spheroid was transferred from the culture plate to an opaque 96-well assay plate (Corning, 3610) prior to assay reagent addition. Spheroid plates were equilibrated at room temperature (RT) for approximately 30 min prior to reagent addition to optimize cell lysis and signal stability. CellTiter-Glo^®^ 3D reagent was added at a 1:1 ratio to the transferred 50 µL spheroid-containing supernatant in each well, and the assay plate was shaken for 5 min at RT at 1,000 rpm to ensure efficient lysis of the 3D structures and thorough mixing of reagent and sample. Plates were incubated for an additional 25 min at RT to allow complete ATP extraction and development of a stable glow-type luminescent signal. Luminescence was measured on a CLARIOstar plate reader (BMG Labtech, 1 s integration time). Sample sizes were *n* = 36 spheroids per handling method for the characterization experiment, and *n* = 12 per handling method and treatment condition for the APAP EC_50_ experiment.

Raw luminescence values were normalized to the cross-sectional area of the corresponding spheroid, as determined by brightfield image analysis performed on the MO:CROSCOPE immediately prior to assay execution (image analysis pipeline described in the Methods section “MO:CROSCOPE, automated brightfield imaging module”) yielding an area-normalized ATP content value per spheroid. For spheroid quality assessment comparing automated (MO:BOT) and manual handling, area-normalized values were expressed as fold changes relative to the manual handling control. For APAP stimulation experiments, area-normalized values were further normalized to DMSO vehicle controls and expressed as a percentage of viability.

### Functional assay: albumin secretion

Albumin secretion, a key marker of liver-specific functionality, was quantified in spheroids generated manually and with the MO:BOT using the Human Albumin ELISA Kit (Abcam, ab108788). Kit components and samples were brought to RT, and albumin standards were prepared by serial dilution according to the manufacturer’s instructions. After thawing and centrifugation (1,500 rpm, 10 min, 4 °C), 50 µL of each supernatant or standard was added to the pre-coated wells and incubated for 1 h at RT. Wells were washed five times with 1X wash buffer, followed by sequential incubations with 50 µL biotinylated anti-albumin antibody and 50 µL streptavidin–HRP conjugate (SP conjugate) for 30 min each, with wash steps between incubations. After a final wash, TMB substrate was added and incubated for 30 min before adding 50 µL of stop solution to each well. Absorbance at 450 nm was measured using a CLARIOstar plate reader for a sample size of *n* = 24 spheroids per handling method.

Albumin concentrations were interpolated from a four-parameter logistic (4PL) standard curve fitted to the serial dilution standards, yielding absolute albumin concentrations expressed as pg/mL of conditioned medium. These values were used to directly compare albumin secretion between manually generated and MO:BOT-generated spheroids.

### Automated acetaminophen (APAP) dose titration

To determine the half-maximal effective concentration (EC₅₀) of acetaminophen (APAP), a dose-response titration experiment was conducted. A 5 M APAP stock solution was prepared in DMSO (Sigma Aldrich, D2438-5 × 10ML) and subsequently diluted in fresh complete DMEM medium to the desired working concentrations ranging from 1 mM to 100 mM. Vehicle control wells received complete DMEM medium containing the corresponding DMSO concentrations without APAP.

The protocol “Liver spheroids APAP dose titration” was selected from the MO:BOT software library. The liver spheroid culture plate was transferred from the incubator, and reagents and consumables were prepared and arranged in the designated MO:BOT work fields. This setup included the liver spheroid culture plate, 1000 µL tips, seven tubes containing culture medium with different APAP concentrations, one tube containing vehicle control medium, and the liquid and tip waste modules. Upon protocol initiation, the MO:BOT automatically acquired a fresh tip, aspirated 80% of the culture medium without disrupting spheroid integrity or 3D morphology, and dispensed the respective APAP treatment solutions according to the predefined plate layout, using new tips between conditions to avoid cross‑contamination.

Following 24 h incubation under standard conditions, spheroid responses were evaluated based on morphological parameters and cell viability. Dose-response curves were generated from normalized viability data, and EC₅₀ values were calculated by nonlinear regression (log[concentration] vs. response, variable-slope model), yielding an EC₅₀ of 40 mM that was subsequently used in downstream experiments. Sample size were *n* = 16 spheroids per APAP concentration.

### Automated medium exchange for EC_50_ APAP stimulation

After establishing 40 mM APAP as the EC₅₀ in liver spheroids, spheroid responses were characterized in both MO:BOT-generated and manually generated spheroids. On day 4 post-seeding, the MO:BOT performed routine 2 of the “Liver spheroids generation and toxicity testing” protocol. The MO:BOT aspirated culture medium from each well and replaced it with either complete DMEM medium containing the corresponding DMSO concentration (vehicle control) or complete DMEM medium containing 40 mM APAP (treatment condition), following the same pipetting workflow described in the Method section “Automated acetaminophen (APAP) dose titration“ but with an adapted number of reagent tubes. Plates were then incubated for 24 h before endpoint analyses were performed. Morphological, functional, and viability readouts were assessed and compared between MO:BOT-generated and manually generated spheroids.

### Cytotoxicity assay in APAP-stimulated liver spheroids

The cytotoxicity of spheroids stimulated with APAP from both handling conditions was analyzed using the LDH-Glo Cytotoxicity Assay (Promega, J2380) following the manufacturer’s instructions. Cell culture plates were equilibrated to RT for approximately 30 min before reagent addition to stabilize LDH activity and luminescent output. For each condition, 50 µL of culture supernatant and 50 µL of LDH-Glo detection reagent were combined in an opaque-walled 96-well plate (Corning, 3610), mixed briefly (500 rpm on a plate shaker), and incubated for 60 min at RT protected from light. Luminescence was measured on a CLARIOstar plate reader (BMG Labtech, 1 s integration time) for a sample size of *n* = 12 spheroids per handling method and treatment condition.

Raw luminescence values were normalized to the cross-sectional area of the corresponding spheroid (µm²), as determined by brightfield image analysis performed on the MO:CROSCOPE immediately prior to assay execution (image analysis pipeline described in the Methods section “MO:CROSCOPE, automated brightfield imaging module”), yielding an area-normalized cytotoxicity value per spheroid. Area-normalized values were expressed as percentage of cytotoxicity relative to a maximum lysis control, enabling direct comparison between untreated and APAP-treated spheroids as well as between MO:BOT-generated and manually generated spheroids.

### Metabolic function: Alanine Aminotransferase (ALT) activity

Hepatocellular toxicity was further evaluated by measuring Alanine Aminotransferase (ALT) activity in culture supernatants using the Human ALT SimpleStep ELISA Kit (Abcam, #ab234578). Reagents, standards, and samples were equilibrated at RT, and the ALT standard curve was prepared according to the kit protocol. For each well, 50 µL of sample or standard, and 50 µL of the antibody cocktail were added, mixed gently, and incubated for 1 h at RT to allow in-well immunocapture. Wells were then washed three times with 1× wash buffer, followed by addition of 100 µL TMB substrate and incubation for approximately 10 min at RT protected from light. The reaction was stopped by adding 100 µL stop solution, and absorbance was measured at 450 nm using a CLARIOStar plate reader. Sample size were *n* = 18 spheroids per handling method.

ALT activity was interpolated from the standard curve and expressed as fold-change relative to the mean of untreated control samples, enabling direct comparison of ALT release between APAP-treated and untreated spheroids. Results from MO:BOT-generated and manually generated spheroids were compared by direct side-by-side visualization.

### MO:CROSCOPE spheroid image segmentation

Spheroid boundaries were delineated using the MIT-B0 SegFormer semantic segmentation model fine-tuned on organoid brightfield images deployed as an ONNX model (ONNX Runtime v1.23.0). Morphological features were computed from the segmented binary masks using OpenCV (v4.11.0) and an in-house Python (Python v3.10) library. Spheroid area was computed as the number of pixels enclosed by the contour boundary and converted to mm² using the calibrated pixel size. Roundness was calculated as the isoperimetric quotient *R = 4πA/P²*, where P is the contour perimeter, yielding *R* = 1 for a perfect circle. Ellipticity was defined as the minor-to-major axis ratio of a least-squares fitted ellipse (*E = a_minor/a_major*), with *E = 1* indicating a circle and lower values indicating elongation.

Prior to quantitative image analysis, all MO:CROSCOPE images were screened for artefacts such as out-of-focus wells, debris, or edge-located spheroids, and wells failing these basic quality checks were excluded from further analysis. Imaging-derived morphological parameters (area, roundness, and ellipticity) were exported from the analysis pipeline described in the Methods section “MO:CROSCOPE, automated bright-field imaging module”, and linked to treatment metadata for downstream statistical comparisons.

### Statistical analysis and manuscript preparation

Group comparisons for functional readouts (viability, cytotoxicity, albumin, ALT) were performed using one-way ANOVA when more than two conditions were compared, followed by Tukey’s HSD post-hoc test to correct for multiple pairwise comparisons. For two group comparisons (e.g., MO:BOT vs. manual at the same treatment condition), unpaired two-tailed Welche’s t-test (unequal variances) was applied after confirming approximate normality and homoscedasticity of residuals. Normality was assessed using the Shapiro-Wilk test and homogeneity of variance using Levene’s test; when assumptions were violated, non-parametric alternatives (Kruskal-Wallis test with Dunn’s post-hoc) were used. Dose-response relationships for APAP titration were modelled by nonlinear regression (log[concentration] vs. response, variable slope) to estimate EC₅₀ values and 95% confidence intervals. The coefficient of variation (CV) values was calculated for each condition as *CV = (SD/mean) × 100* across biological replicates to assess assay variability and robustness. Statistical significance was evaluated with a p value < 0.05 considered statistically significant. Significance levels: *p < 0.05, **p < 0.01, ***p < 0.001, ****p < 0.0001.

A large language model-based assistant (Perplexity, powered by GPT-5.1) was used to support manuscript text editing and phrasing. All scientific content, data interpretation, and final text were reviewed and approved by the authors.

## Supplementary Information

Below is the link to the electronic supplementary material.

Supplementary Material 1

Supplementary Material 2

## Data Availability

The datasets generated and analyzed during the current study are available from the corresponding authors on reasonable request.
