# Supplementary material for: Automation of 3D liver spheroid generation and acetaminophen dose–response on the MO:BOT enhances assay robustness and precision
Source: Sci Rep. 2026 Jul 21;16:22707. doi: 10.1038/s41598-026-58939-4 (PMC13388963; doi:10.1038/s41598-026-58939-4)
Supplement: Supplementary file 1 — Supplementary Material 1 [file 41598_2026_58939_MOESM1_ESM.pdf]

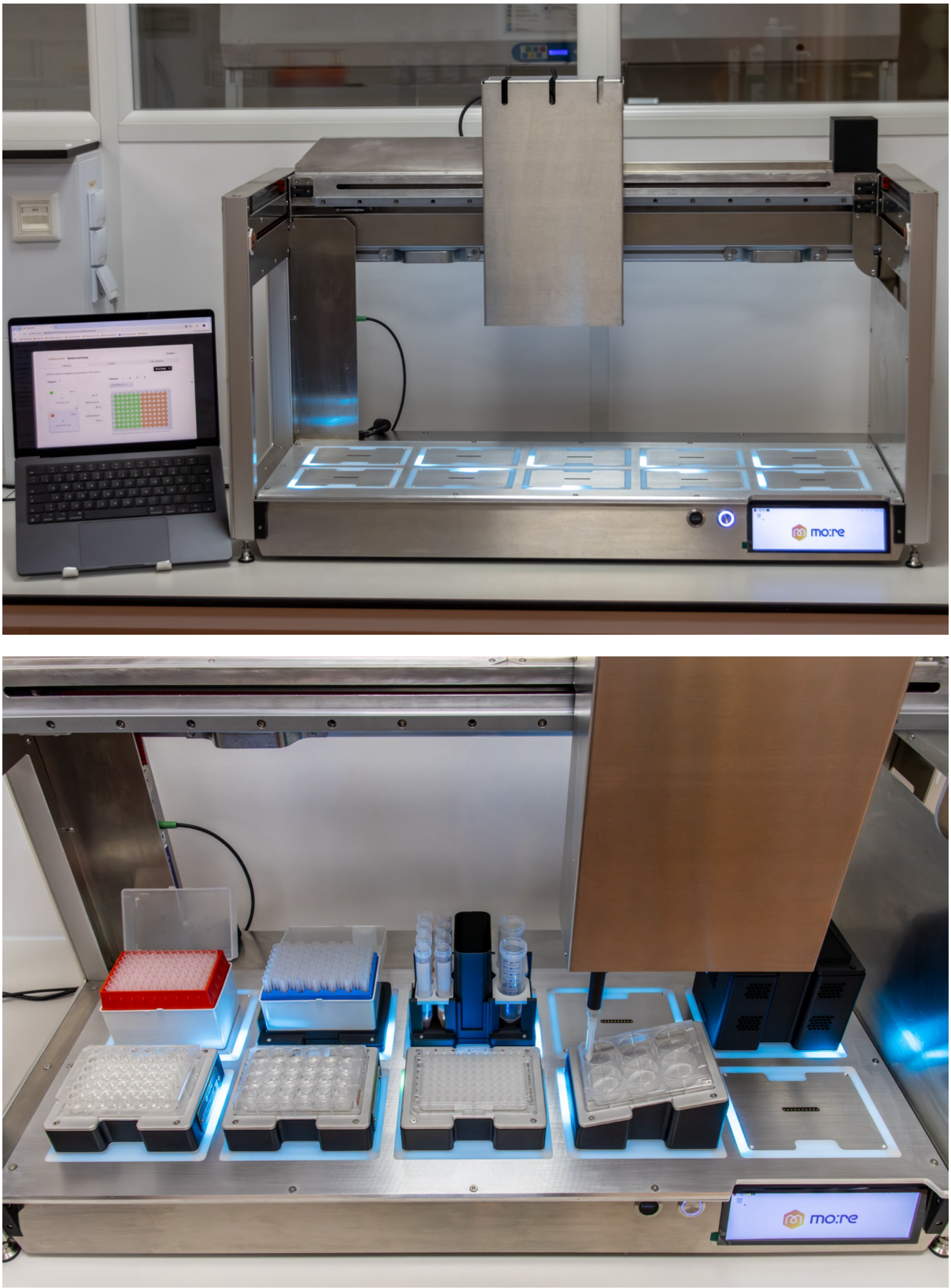

Supplementary Figure 1: Representative photographs of the MO:BOT without integrated modules (top) and with modules install (bottom).

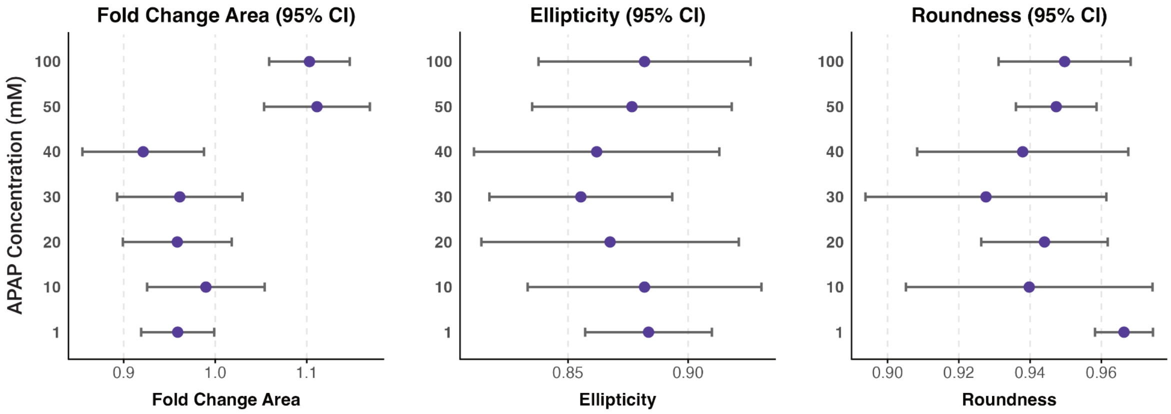

Supplementary Figure 2: Fold change area (left), ellipticity (centre), and roundness (right) of liver spheroids following exposure to acetaminophen (APAP) at indicated concentrations. Points represent means; error bars indicate 95% confidence intervals. Only fold change area at APAP concentrations of 100 and 50 mM show a meaningful difference relative to concentrations of 1 – 40 mM.
